# Supplementary material for: Dietary Acrylamide Intake Alters Gut Microbiota in Mice and Increases Its Susceptibility to Salmonella Typhimurium Infection
Source: Foods. 2021 Dec 3;10(12):2990. doi: 10.3390/foods10122990 (PMC8700958; doi:10.3390/foods10122990)
Supplement: Supplementary file 1 [file foods-10-02990-s001.zip › foods-1464232-supplementary.pdf]

*Supplementary materials*

# Dietary acrylamide intake alters gut microbiota in mice and increases its susceptibility to Salmonella Typhimurium infection

**Table S1.** Primer sequences.

| Gene | Forward primer | Reverse primer |
|---|---|---|
| *IL-6* | 5′-GCTACCAAACTGGATA-TAATCAGGA-3′ | 5′-CCAGGTAGCTATGG-TACTCCAGAA-3′ |
| *IL-1β* | 5′-TTGACGGACCCCAAAAGAT-3′ | 5′-AGCTGGATGCTCTCATCAGG-3′ |
| *TNF-α* | 5′-TCTTCTCATTCCTGCTTGTGG-3′ | 5′-GGTCTGGGCCATAGAACTGA-3′ |
| *Mucin2* | 5′-TCATGACGTCTGGTGGAATGG-3′ | 5′-AGCTTGTCGTGGCAGCTTGA-3′ |
| *E-cadherin* | 5′-GCCAATCCTGATGAAATT-GGAAAC-3′ | 5′-CCACTGCCCTCGTAATCGAAC-3′ |
| *Occludin* | 5′-GGCAAGCGATCATACCCAGAG-3′ | 5′-AGGCTGCCTGAAGTCATCCAC-3′ |
| *ZO-1* | 5′-GACCAATAGCTGATGTT-GCCAGAG-3′ | 5′-TATGAAGGCGAATGATGCCAGA-3′ |
| *Claudin-1* | 5′-AGTGCATGAGGTGCCTGGAAG-3′ | 5′-TGGCCACTAATGTCGCCAGA-3′ |
| *GAPDH* | 5′-ACAGTCCATGCCATCACTGCC-3′ | 5′-GCCTGCTTCACCACCTTCTTG-3′ |

**Table S2.** Diversity and richness of fecal microbiota in NC and AA groups

| Groups | Richness | | Diversity | | Observed_Species | Goods_Coverage |
|---|---|---|---|---|---|---|
| | Chao1 | ACE | Shannon | Simpson | | |
| Control | 487.977±36.948[a] | 475.698±38.079[a] | 5.886±0.133[a] | 0.963±0.005[a] | 375.500±6.985[a] | 0.999±0.0001[a] |
| AA | 334.236±47.104[b] | 326.063±41.678[b] | 5.743±0.130[a] | 0.960±0.004[a] | 255.333±11.885[b] | 0.999±0.0001[a] |

The means with different superscript letters (a and b) respresent statistically signinficant results ($p < 0.001$) based on Two-way ANOVA with Bonferroni multiple comparisons test, whereas means labeled with the same superscript correspond to results that show no statistically significant differences. The results were shown as means ± SEM.

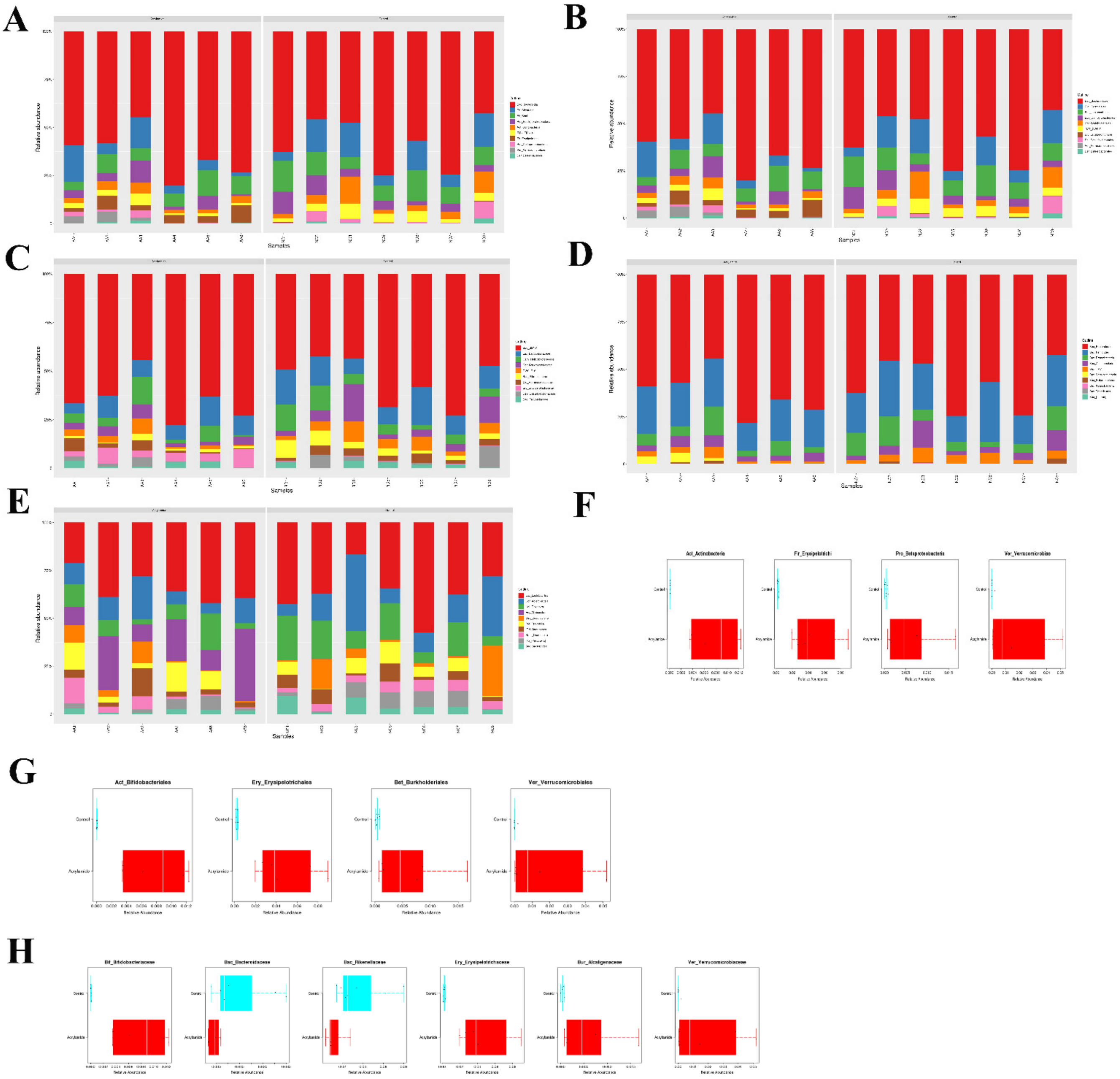


**Figure S1.** The difference analysis of fecal microbiota between two groups in different levels. (A) Relative abundance of fecal microbiota in each sample at class level. (B) Relative abundance of fecal microbiota in each sample at order level. (C) Relative abundance of fecal microbiota in each sample at family level. (D) Relative abundance of fecal microbiota in each sample at phylum level. (E) Relative abundance of fecal microbiota in each sample at genus level. (F) Comparison of relative abundance of significant bacteria taxa between two groups at class level. (G) Comparison of relative abundance of significant bacteria taxa between two groups at order level. (H) Comparison of relative abundance of significant bacteria taxa between two groups at family level.
